# Foetal Reduction in a Cornual Heterotopic Pregnancy With Potassium Chloride Administration

**DOI:** 10.7759/cureus.104535

**Published:** 2026-03-02

**Authors:** Yi Hui Liu

**Affiliations:** 1 Obstetrics and Gynaecology, St George Hospital, Sydney, AUS

**Keywords:** 1. high risk obstetrics 2. assisted reproduction 3. advanced endoscopic surgery 4. reproductive endocrinology 5. foetal medicine 6. gynaecological oncology, gynaecology and obstetrics, obs & gynae, obstetrics & gynecology, obstetrics ultrasonography

## Abstract

Heterotopic pregnancy is the simultaneous occurrence of an intrauterine pregnancy with an extrauterine pregnancy. Heterotopic pregnancies remain a diagnostic challenge, as many can be asymptomatic, or clinicians could be falsely reassured once an intrauterine pregnancy is sighted on an ultrasound. Given their rarity, there is also no global consensus on management guidelines. In this study, we illustrate a case of a heterotopic pregnancy with preservation of the intrauterine pregnancy and management of the ectopic pregnancy with potassium chloride (KCl) administration.

## Introduction

Heterotopic pregnancy is a rare condition in which there is the simultaneous conception of an intrauterine and an ectopic pregnancy. The incidence is reportedly 1 in 30,000 spontaneous conceptions but increases to as high as 1 in 100 pregnancies in patients undergoing assisted reproductive technology (ART) [1,2]. The significant difference in incidence is likely secondary to multiple embryo transfers in ART [3,4]. The most common extrauterine sites include the fallopian tubes, which account for approximately 90% of all heterotopic pregnancies, and less commonly cornual, cervical, caesarean, and abdominal sites [5]. Heterotopic pregnancies typically manifest with abdominal pain, peritoneal irritation, and vaginal bleeding, but a minority of patients can be asymptomatic. Heterotopic pregnancies remain a diagnostic challenge, as symptoms can range widely, and the presence of a confirmed intrauterine pregnancy can delay diagnosis [6]. In this study, we present a case of a naturally conceived heterotopic pregnancy and the management of the ectopic pregnancy using KCl with conservation of the intrauterine viable pregnancy.

## Case presentation

**Figure 1:**
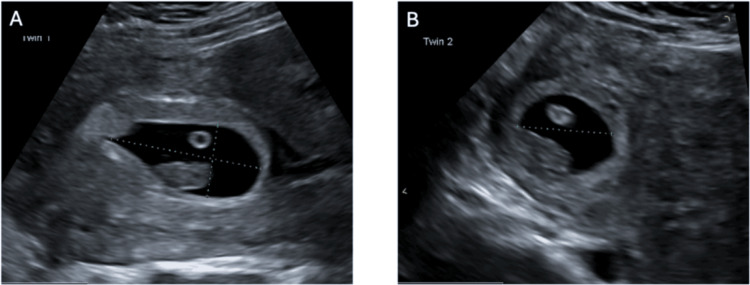
Transvaginal ultrasound demonstrating a heterotopic pregnancy (A) Intrauterine pregnancy with a gestational sac corresponding to approximately 7+5 weeks gestation. (B) Cornual ectopic pregnancy with a gestational sac corresponding to approximately 7+5 weeks gestation.

The patient is a female in her 40s, gravidarum 2 parity 1, who was sent in from an outpatient ultrasound facility to the emergency department with a confirmed heterotopic pregnancy at approximately seven weeks of gestation (Figure 1). The ultrasound was initially completed as a dating scan. She was haemodynamically stable and asymptomatic, including nil abdominal pain. Her blood results were unremarkable with a BHCG of 189,910 IU/L.

Her obstetric history included one previous elective lower segment caesarean section in 2022. She had regular 28-day menstrual cycles. Her gynaecological history was significant for a known multifibroid uterus with a previous laparoscopic myomectomy in 2013 for an intramural fibroid. She had a history of genital herpes with no flare-ups in the last few years. She had an up-to-date cervical screening test and nil history of sexually transmitted infections or pelvic inflammatory diseases. Her past medical history was significant for Hashimoto's thyroiditis, asthma, and mild mitral regurgitation, which she regularly follows up with a cardiologist. Her regular medications include 50 micrograms of thyroxine daily and Symbicort (budesonide-formoterol) and salbutamol as needed. She had no known drug allergies.

The impression is a heterotopic cornual pregnancy with a viable intrauterine pregnancy. The management options were provided to the patient for either preservation of the intrauterine pregnancy with ultrasound-guided KCl injection into the cornual ectopic pregnancy or laparoscopic removal of the cornual ectopic pregnancy, or termination of both the cornual and intrauterine pregnancies with laparoscopic removal of the ectopic pregnancy, a dilatation of the cervix, and curettage of the uterus.

**Figure 2:**
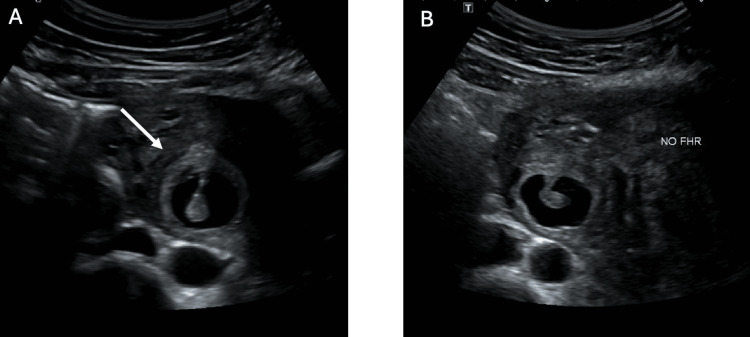
Ultrasound-guided administration of potassium chloride (KCl) (A) Ultrasound demonstrating disruption of the gestational sac and injection of the KCl. (B) Ultrasound post-administration of KCl demonstrating subsequent asystole in the cornual ectopic pregnancy.

**Figure 3:**
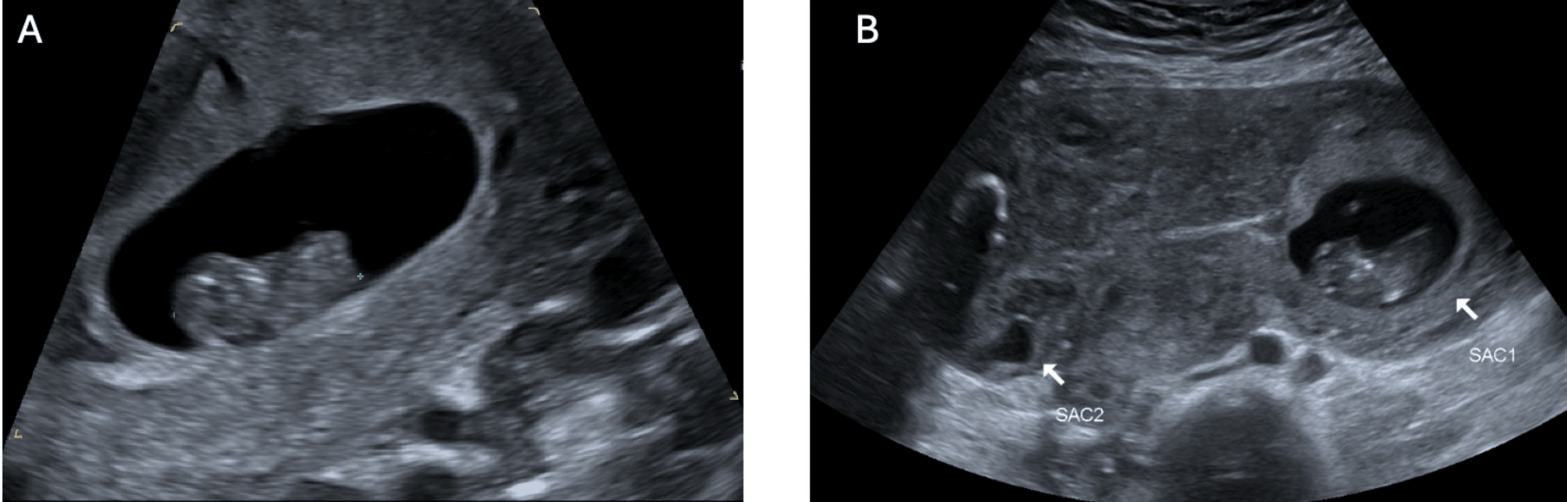
Pelvic ultrasound seven days post KCl administration (A) Progressive intrauterine pregnancy that is approximately 9+2 gestation by crown rump length. (B) Sac 1 illustrating progressive intrauterine pregnancy and Sac 2 demonstrating previous right cornual ectopic pregnancy with nil further growth. KCl: Potassium chloride

**Figure 4:**
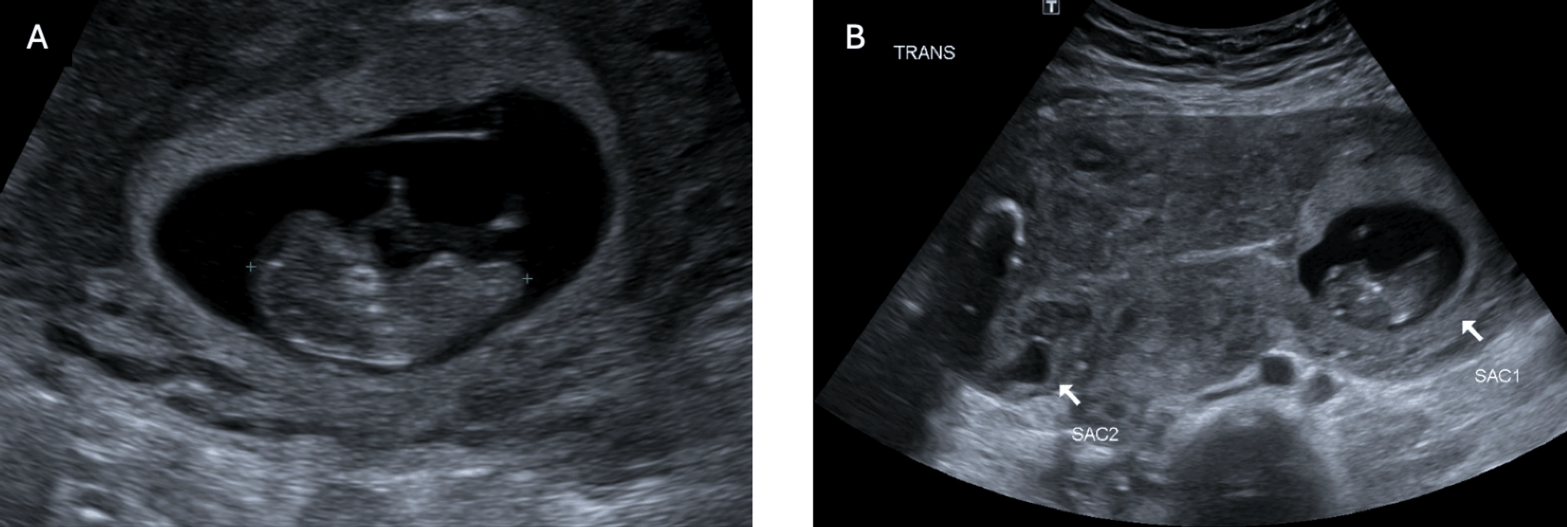
Pelvic ultrasound 14 days post KCl administration (A) Progressive intrauterine pregnancy that is approximately 10+1 weeks gestation by crown rump length. (B) Sac 1 illustrating progressive intrauterine pregnancy and Sac 2 demonstrating previous right cornual ectopic pregnancy with nil further growth (approximately 1.0 x 8.0 x 1.2 cm).

**Figure 5:**
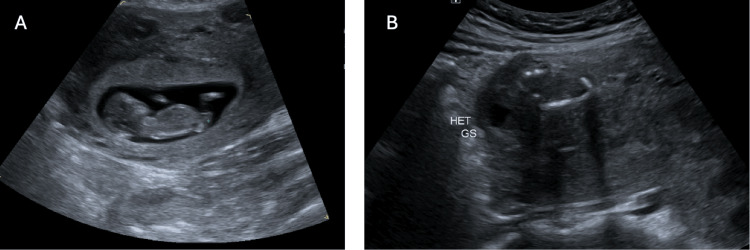
Pelvic ultrasound 21 days post-KCl administration (A) Progressive intrauterine pregnancy that is approximately 11+4 weeks gestation by crown rump length. (B) Previous right cornual ectopic pregnancy with nil further growth (approximately 1.7 x 0.9 x 1.1 cm).

**Figure 6:**
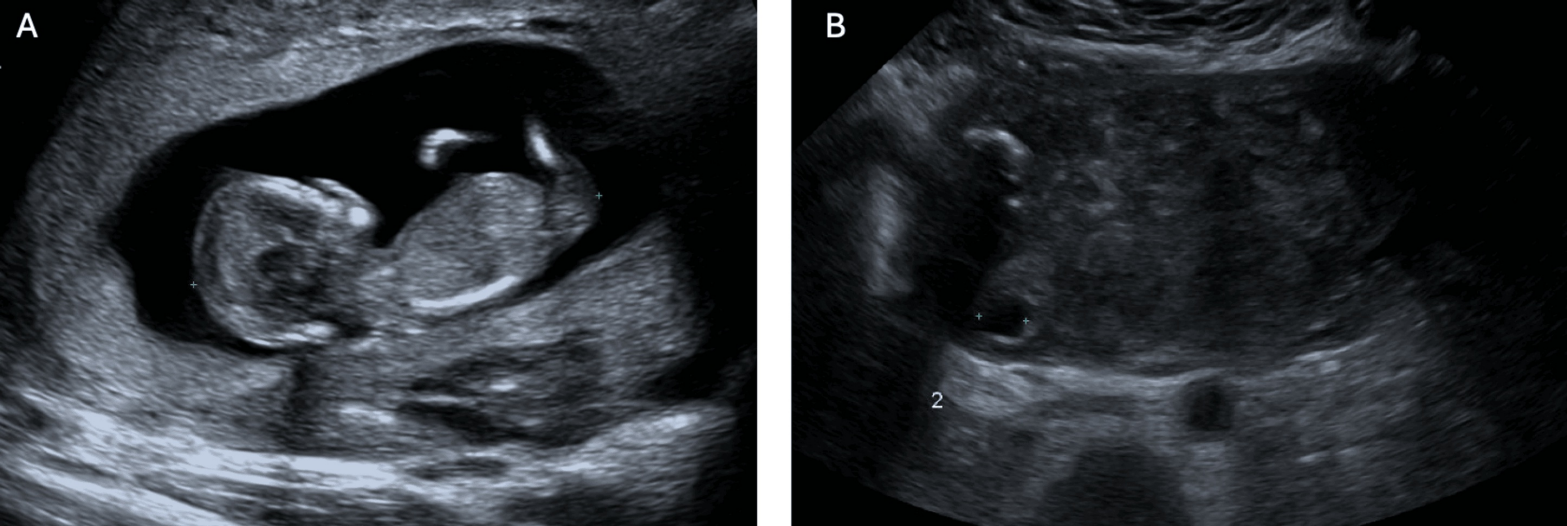
Pelvic ultrasound 28 days post-KCl administration (A) Progressive intrauterine pregnancy that is approximately 13+3 weeks gestation by crown rump length. (B) Previous right cornual ectopic pregnancy with nil further growth (1.4 x 1.1 x 0.9 cm).

A shared decision was made for ultrasound-guided KCl injection into the cornual ectopic pregnancy with attempted preservation of the intrauterine pregnancy. The procedure was completed with a 22-gauge needle into the ectopic gestational sac with mechanical disruption of the foetal pole, 5 mL of the gestational sac fluid aspirated, and 0.75 g of potassium chloride injected into the foetal pole/gestational sac. A short-interval progress ultrasound after the procedure demonstrated a foetal heartbeat in the intrauterine pregnancy and no foetal heartbeat in the extrauterine pregnancy, presuming foetal demise (Figure 2). The patient was well and able to go home the same day. She was followed up through the early pregnancy outpatient service with weekly pelvic ultrasounds, demonstrating a progressive viable intrauterine pregnancy with the demise of the cornual ectopic pregnancy (Figure 3-6). Since the time of publication, she continues to have a progressive pregnancy with regular antenatal care.

## Discussion

This case contributes to the growing literature on heterotopic pregnancies while also demonstrating two important points. Heterotopic pregnancies remain a diagnostic challenge for clinicians, as they could manifest asymptomatically or in vague symptoms that often occur in early pregnancy. There could also be a delay in diagnosis with false reassurance when ultrasounds confirm an intrauterine pregnancy. Thus, it is important for clinicians to have a high degree of suspicion and be aware of the risk factors for heterotopic pregnancy. These risk factors include assisted reproductive technologies, particularly in vitro fertilisation and embryo transfer, tubal pathology, history of pelvic inflammatory disease, history of multiple abortions, previous abdomino-pelvic or tubal surgery, and family history of multiple gestations [3,7-10]. In this study, we demonstrate a patient with some risk factors, including a history of previous laparoscopic myomectomy and a previous caesarean section. However, she did not have any other risk factors, including previous tubal pathology, did not have any classic symptoms, and had naturally conceived without the use of ART. The diagnosis was made from a routine antenatal dating scan at a tertiary centre. Thus, it is important for clinicians to be aware of this rare but serious condition and to consider it as a possible differential diagnosis for any pregnant woman.

Second, this case shares a unique management process for a heterotopic pregnancy to preserve a viable intrauterine pregnancy. The current mainstay management of a heterotopic pregnancy is surgical removal of the extrauterine gestation [11,12]. However, with the advent of readily accessible ultrasound imaging and multidisciplinary teams with interventional radiologists, there should be further consideration of the use of medical management with KCl. The advantages of medical management over surgical management include faster recovery, reduction in blood loss, shorter hospital stays, and less invasive procedures. Previous studies have demonstrated more favourable outcomes for surgical management of cornual heterotopic pregnancies, with only half of those with KCl administration achieving a live birth [13]. However, this is more likely due to the lack of patient risk stratification. In stable patients with early detection of a heterotopic pregnancy and low risk of abscondment, there is less risk of rupture; thus, medical management can arguably be more beneficial than surgical management. Fernandez et al. developed a scoring system that evaluated the success of non-surgical management with heterotopic cornual pregnancies by looking at gestational sac, beta-human chorionic gonadotrophin level, progesterone level, presence of abdominal pain, volume of hemoperitoneum, and ultrasonographic diameter of hematosalpinx. This study found that if the total score was ≤12, then the likelihood of success of medical treatment was >80% [14]. This case demonstrates the same principles in which a stable patient with an early diagnosis was able to achieve selective foetal reduction with KCl and continue with a progressive pregnancy.

Currently, there is no consensus for the treatment of heterotopic pregnancies. Further large-scale studies will need to be completed to demonstrate maternal and foetal outcomes that may help further guide management in these cases and to differentiate parameters in which medical management may be more advantageous than surgical management. 

## Conclusions

Heterotopic pregnancies are rare but serious conditions that clinicians should be aware of and rule out in early pregnancy, particularly with the increase in incidence with ART. This case illustrates a patient who was able to have a successful reduction of an ectopic pregnancy with preservation of the intrauterine pregnancy using KCl. Simultaneously, this case also illustrates the need for further research to help develop international guidelines on the management of heterotopic pregnancies.
